# Analysis of the Most Frequently Cited Articles in Hand and Wrist Surgery: A Modern Reading List

**DOI:** 10.7759/cureus.32690

**Published:** 2022-12-19

**Authors:** Stephanie Delma, Yagiz Ozdag, Eugene P Warnick, Jessica Baylor, Louis C Grandizio

**Affiliations:** 1 Orthopaedics, Geisinger Commonwealth School of Medicine, Danville, USA

**Keywords:** orthopaedic surgery, bibliometric analysis, citations, wrist surgery, hand surgery

## Abstract

The aim of this investigation was to analyze the 50 most frequently cited articles on hand and wrist surgery of all time and those published during the 21^st^ century. We aimed to evaluate the article, author, and journal characteristics for these publications in order to create a modern reading list of impactful upper-extremity articles.

A search of the Journal Citation Reports 2022 edition to identify journals containing possible hand or wrist-related articles was performed. Related journals were identified and then searched on the Web of Science database to identify hand and wrist articles. The top 50 most cited articles overall and the top 50 most cited articles from 2000-2021 were identified and indexed. Several bibliometric parameters, such as study type, study topic, study design, level of evidence, citation count, citation density, the institution of the lead author, the gender of lead and senior authors, and country were analyzed.

For the most cited articles of all time, the number of citations ranged from 224 to 1109 with a mean of 368 citations and 15.0 citation density. Citations for the top 50 articles from 2000-2021 ranged from 153 to 950 with a mean of 233 citations and 14.5 citation density. For both groups, the most common level of evidence was level IV (33% and 27%). No correlation between journal impact factor and citation count or citation density was found. In both groups, “fracture” was the most common topic and papers were predominantly written by male authors.

Frequently cited publications on hand and wrist surgery are often clinical papers that contain low levels of evidence and tend to focus on topics related to fracture care. Female authors remain underrepresented.

## Introduction and background

There has been a substantial increase in the volume of peer-reviewed academic articles published during the past few decades across a variety of medical subspecialties [1]. Recent investigations have indicated that there were over 8,000 orthopedic surgery articles published between 2010 and 2014 from US-based institutions [2]. As article volume has continued to increase, so too has the number of orthopedic surgery journals. A recent publication reported that there were around 300 indexed orthopedic surgery journals as of 2020 under the Scimago database [3]. As both journal and article volumes continue to increase, upper-extremity surgeons may face challenges in navigating the available literature to identify impactful and relevant articles [3-6]. “Classic” papers have helped build the foundation of modern practices by describing and analyzing the basic science concepts, techniques, outcomes, and complications of commonly performed procedures. While a variety of metrics are available to quantify an individual paper’s influence, the most common and convenient way continues to be the number of citations the scientific work has received [7]. In this context, bibliometric analyses of top-cited articles can aid in understanding the characteristics of classic papers.

The purpose of this investigation was to identify and analyze the 50 most frequently cited articles on hand and wrist surgery. We aimed to evaluate the article, author, and journal characteristics for these frequently cited publications. In addition to identifying the most cited articles of all time, we aimed to define the most frequently cited hand and wrist articles published in the 21^st^ century in order to create a modern reading list of impactful upper-extremity articles.

## Review

Materials & methods

Search Criteria

In February 2022, we conducted a search of the Journal Citation Reports 2022 edition to identify journals containing possible hand or wrist-related articles. The Journal Citation Reports, published annually by Clarivate Analytics, is a database of basic science and social science academic journals [8]. This database provides tools to identify, categorize, and compare journals based on journal topics and citation metrics. We identified journals under the categories, “ORTHOPEDICS”, “SPORTS SCIENCES”, and “SURGERY” and refined the search with the terms “hand OR wrist”. Journals were then searched on the Web of Science database to identify hand and wrist articles. The Web of Science is a Clarivate Analytics database that continually indexes and updates information on scholarly articles [8]. The platform allows users to search articles by journal or topic and track an article's citations or cited references. The top 1000 most cited articles were exported from the Web of Science database and then sorted by “times cited” in descending order. The articles were then reviewed by two authors for inclusion criteria (whether the identified articles were pertinent to orthopedic hand and wrist surgery) from the most to least cited until 50 articles were included. Articles were excluded if they did not focus on the surgery of the hand or wrist.

Data Extraction

From the remaining articles, the top 50 most cited articles overall and the top 50 most cited articles from 2000-2021 were determined. For all included articles, data from the Web of Science database for the following items were included: title, lead author, senior author, journal name, number of citations, citation density, and publication year. Citation density is determined by dividing the total number of citations by the number of years the article has been published. Additionally, two reviewers reviewed articles and recorded the study type (clinical, basic science), study topic (fracture, osteoarthritis, outcomes, etc.), study design, level of evidence, the institution of the lead author, gender of the lead and senior authors, and country. For the purposes of our investigation, we used the last name in the author list of each article to designate senior author status. None of the authors of this analysis were authors of the articles that were indexed in our investigation. The level of evidence was assigned according to the Journal of Bone & Joint Surgery guidelines (see Appendices) [9]. Where the gender of the author was undiscernible, the Genderize.io API was used to determine gender by first name [10]. Any disagreements in the classification of articles were reconciled by a third reviewer. Any disagreements in the classification of articles were reconciled by a third reviewer. Overall, there were only five disagreements where the initial reviewers could not settle on the level of evidence of an article. The 2020 two-year impact factor was obtained from each journal’s respective web page.

Statistical Analysis

Descriptive statistics were measured to summarize the results. Means and percentages were used to compare articles from all years and articles from 2000-2022. A chi-squared test of independence analysis was used to compare author gender between groups. P-values < 0.05 were considered significant. All data analyses were conducted using IBM SPSS Statistics Version 28.0.2.2(15) (IBM Corp., Armonk, NY).

Results

The top 50 most cited hand and wrist articles overall and from the 21^st^ century are compiled in Tables 1-2. For the most cited articles of all time, the number of citations ranged from 224 to 1109 with a mean of 368 citations and 15.0 citation density. The oldest article was published in 1972 and the most recent was published in 2014 with 555 citations and 272 citations, respectively.

**Table 1:** Top 50 most cited articles

Rank	Article	# of Citations (Citation Density)
1	Mathiowetz V, Weber K, Volland G, et al. Reliability and validity of grip and pinch strength evaluations. J Hand Surg Am. 1984;9(2):222-226.	1109 (29.2)
2	Beaton DE, Wright JG, Katz JN, et al. Development of the QuickDASH: comparison of three item-reduction approaches. J Bone Joint Surg Am. 2005;87(5):1038-1046.	950 (55.9)
3	Chung KC, Pillsbury MS, Walters MR, et al. Reliability and validity testing of the Michigan Hand Outcomes Questionnaire. J Hand Surg Am. 1998;23(4):575-587.	629 (26.2)
4	Palmer AK. Triangular fibrocartilage complex lesions: a classification. J Hand Surg Am. 1989;14(4):594-606.	625 (18.9)
5	Linscheid R, Dobyns J, Beabout J, et al. Traumatic instability of the wrist. J Bone Joint Surg Am. 1972;54(8):1612-1632.	555 (11.1)
6	Palmer A, Werner F. The triangular fibrocartilage complex of the wrist—anatomy and function. J Hand Surg Am. 1981;6(2):153-162.	537 (13.1)
7	Watson HK, Ballet FL. The SLAC wrist: scapholunate advanced collapse pattern of degenerative arthritis. J Hand Surg Am. 1984;9(3):358-365.	527 (13.9)
8	MacDermid J, Turgeon T, Richards R, et al. Patient Rating of Wrist Pain and Disability: A Reliable and Valid Measurement Tool. J Orthop Trauma. 1998;12(8):577-586.	499 (20.8)
9	Eaton RG, Littler JW. Ligament reconstruction for the painful thumb carpometacarpal joint. J Bone Joint Surg Am. 1973;55(8):1655-1666.	490 (10)
10	Arora R, Lutz M, Hennerbichler A, et al. Complications following internal fixation of unstable distal radius fracture with a palmar locking-plate. J Orthop Trauma. 2007;21(5):316-322.	424 (28.3)
11	Weber RA, Breidenbach WC, Brown RE, et al. A randomized prospective study of polyglycolic acid conduits for digital nerve reconstruction in humans. Plast Reconstr Surg. 2000;106(5):1036-8.	398 (18.1)
12	Strickland JW, Glogovac SV. Digital function following flexor tendon repair in Zone II: A comparison of immobilization and controlled passive motion techniques. J Hand Surg Am. 1980;5(6):537-543.	395 (9.4)
13	Burton RI, Pellegrini VD. Surgical management of basal joint arthritis of the thumb. Part II. Ligament reconstruction with tendon interposition arthroplasty. J Hand Surg Am. 1986;11(3):324-332.	383 (10.6)
14	Youm Y, McMurthy RY, Flatt AE, et al. Kinematics of the wrist. I. An experimental study of radial-ulnar deviation and flexion-extension. J Bone Joint Surg Am. 1978;60(4):423-431.	374 (8.5)
15	Chung K, Spilson S. The frequency and epidemiology of hand and forearm fractures in the United States. J Hand Surg Am. 2001;26(5):908-915.	373 (17.8)
16	Trumble T, Schmitt S, Vedder N. Factors affecting functional outcome of displaced intra-articular distal radius fractures. J Hand Surg Am. 1994;19(2):325-340.	360 (12.9)
17	Mayfield JK, Johnson RP, Kilcoyne RK. Carpal dislocations: pathomechanics and progressive perilunar instability. J Hand Surg Am. 1980;5(3):226-241.	359 (8.5)
18	Orbay J, Fernandez D. Volar fixation for dorsally displaced fractures of the distal radius: A preliminary report. J Hand Surg Am. 2002;27(2):205-215.	358 (17.9)
19	Geissler WB, Freeland AE, Savoie FH, et al. Intracarpal soft-tissue lesions associated with an intra-articular fracture of the distal end of the radius. J Bone Joint Surg Am. 1996;78(3):357-365.	355 (13.7)
20	Agee JM, McCarroll HR, Tortosa RD, et al. Endoscopic release of the carpal tunnel: a randomized prospective multicenter study. J Hand Surg Am. 1992;17(6):987-995.	344 (11.5)
21	Cooney WP, Chao EY. Biomechanical analysis of static forces in the thumb during hand function. J Bone Joint Surg Am. 1977;59(1):27-36.	343 (7.6)
22	Palmer A, Werner F, Murphy D, et al. Functional wrist motion: A biomechanical study. J Hand Surg Am. 1985;10(1):39-46.	335 (9.1)
23	Eaton R, Glickel S. Trapeziometacarpal Osteoarthritis. Hand Clin. 1987;3(4):455-469.	322 (9.2)
24	Schuind F, Garcia-Elias M, Cooney W, et al. Flexor tendon forces: In vivo measurements. J Hand Surg Am. 1992;17(2):291-298.	322 (10.7)
25	Brand P, Beach R, Thompson D. Relative tension and potential excursion of muscles in the forearm and hand. J Hand Surg Am. 1981;6(3):209-219.	318 (7.8)
26	Orbay JL, Fernandez DL. Volar fixed-angle plate fixation for unstable distal radius fractures in the elderly patient. J Hand Surg Am. 2004;29(1):96-102.	318 (17.7)
27	Brown R, Gelberman R, Seiler J et al. Carpal tunnel release. A prospective, randomized assessment of open and endoscopic methods. J Bone Joint Surg Am. 1993;75(9):1265-1275.	311 (10.7)
28	Mintken PE, Glynn P, Cleland JA. Psychometric properties of the shortened disabilities of the Arm, Shoulder, and Hand Questionnaire (QuickDASH) and Numeric Pain Rating Scale in patients with shoulder pain. J Shoulder Elbow Surg. 2009;18(6):920-926.	301 (23.2)
29	Nellans K, Kowalski E, Chung K. The Epidemiology of Distal Radius Fractures. Hand Clin. 2012;28(2):113-125.	300 (30.0)
30	Amadio P, Berquist T, Smith D, et al. Scaphoid malunion. J Hand Surg Am. 1989;14(4):679-687.	295 (8.9)
31	Rozental TD, Blazar PE. Functional outcome and complications after volar plating for dorsally displaced, unstable fractures of the distal radius. J Hand Surg Am. 2006;31(3):359-365.	291 (18.2)
32	Zaidemberg C, Siebert JW, Angrigiani C. A new vascularized bone graft for scaphoid nonunion. J Hand Surg Am. 1991;16(3):474-478.	289 (9.3)
33	Crosby CA, Wehbe MA, Mawr B. Hand strength: normative values. J Hand Surg Am. 1994;19(4):665-670.	286 (10.2)
34	Petruzzo P, Lanzetta M, Dubernard JM, et al. The International Registry on Hand and Composite Tissue Transplantation. Transplantation. 2010;90(12):1590-1594.	284 (23.7)
35	Arora R, Lutz M, Deml C, et al. A Prospective Randomized Trial Comparing Nonoperative Treatment with Volar Locking Plate Fixation for Displaced and Unstable Distal Radial Fractures in Patients Sixty-five Years of Age and Older. J Bone Joint Surg Am. 2011;93(23):2146-2153. 7	280 (25.5)
36	Palmer A, Glisson R, Werner F. Ulnar variance determination. J Hand Surg Am. 1982;7(4):376-379.	276 (6.9)
37	Franchignoni F, Vercelli S, Giordano A, et al. Minimal Clinically Important Difference of the Disabilities of the Arm, Shoulder and Hand Outcome Measure (DASH) and Its Shortened Version (QuickDASH). J Orthop Sports Phys. 2014;44(1):30-39.	272 (34)
38	MacDermid J, Richards R, Donner A, et al. Responsiveness of the short form-36, disability of the arm, shoulder, and hand questionnaire, patient-rated wrist evaluation, and physical impairment measurements in evaluating recovery after a distal radius fracture. J Hand Surg Am. 2000;25(2):330-340.	272 (12.4)
39	Chung KC, Shauver MJ, Birkmeyer JD. Trends in the United States in the treatment of distal radial fractures in the elderly. J Bone Joint Surg Am. 2009;91(8):1868-1873.	271 (20.8)
40	Gelberman R, Menon J. The vascularity of the scaphoid bone. J Hand Surg Am. 1980;5(5):508-513.	268 (6.4)
41	Ryu JY, Cooney WP, Askew LJ, et al. Functional ranges of motion of the wrist joint. J Hand Surg Am. 1991;16(3):409-419.	256 (8.3)
42	Hoff B, Arbib M. Models of Trajectory Formation and Temporal Interaction of Reach and Grasp. J Mot Behav. 1993;25(3):175-192.	252 (8.7)
43	Rantanen T, Masaki K, Foley D, et al. Grip strength changes over 27 yr in Japanese-American men. J Appl Physiol. 1998;85(6):2047-2053.	247 (10.3)
44	Lieber R, Jacobson M, Fazeli B, et al. Architecture of selected muscles of the arm and forearm: Anatomy and implications for tendon transfer. J Hand Surg Am. 1992;17(5):787-798.	243 (8.1)
45	Eaton RG, Lane LB, Littler JW, et al. Ligament reconstruction for the painful thumb carpometacarpal joint: a long-term assessment. J Hand Surg Am. 1984;9(5):692-699.	242 (6.4)
46	Herzberg G, Comtet J, Linscheid R, et al. Perilunate dislocations and fracture-dislocations: A multicenter study. J Hand Surg Am. 1993;18(5):768-779.	241 (8.3)
47	Coleman S. Hand Rejuvenation with Structural Fat Grafting. Plast Reconstr Surg. 2002;110(7):1731-1744.	237 (11.9)
48	Buchthal F, Rosenfalck A, Trojaborg W. Electrophysiological findings in entrapment of the median nerve at wrist and elbow. J Neurol Neurosurg Psychiatry. 1974;37(3):340-360.	236 (4.9)
49	Peolsson, Rune Hedlund, Birgitta Ob A. Intra- and inter-tester reliability and reference values for hand strength. J Rehabil Med. 2001;33(1):36-41.	236 (11.2)
50	Dhillon G, Lawrence S, Hutchinson D, et al. Residual function in peripheral nerve stumps of amputees: implications for neural control of artificial limbs. J Hand Surg Am. 2004;29(4):605-615.	224 (12.4)

**Table 2:** Top 50 most cited articles since 2000

Rank	Total Citation	# of Citations (Citation Density)
1	Beaton DE, Wright JG, Katz JN, et al. Development of the QuickDASH: comparison of three item-reduction approaches. J Bone Joint Surg Am. 2005;87(5):1038-1046.	950 (55.9)
2	Arora R, Lutz M, Hennerbichler A, et al. Complications following internal fixation of unstable distal radius fracture with a palmar locking-plate. J Orthop Trauma. 2007;21(5):316-322.	424 (28.3)
3	Weber RA, Breidenbach WC, Brown RE, Jabaley ME, Mass DP. A randomized prospective study of polyglycolic acid conduits for digital nerve reconstruction in humans. Plast Reconstr Surg. 2000;106(5):1036-1038.	398 (18.1)
4	Chung K, Spilson S. The frequency and epidemiology of hand and forearm fractures in the United States. J Hand Surg Am. 2001;26(5):908-915.	373 (17.8)
5	Orbay JL, Fernandez DL. Volar fixation for dorsally displaced fractures of the distal radius: A preliminary report. J Hand Surg Am. 2002;27(2):205-215.	358 (17.9)
6	Orbay JL, Fernandez DL. Volar fixed-angle plate fixation for unstable distal radius fractures in the elderly patient. J Hand Surg Am. 2004;29(1):96-102.	318 (17.7)
7	Mintken PE, Glynn P, Cleland JA. Psychometric properties of the shortened disabilities of the Arm, Shoulder, and Hand Questionnaire (QuickDASH) and Numeric Pain Rating Scale in patients with shoulder pain. J Shoulder Elbow Surg. 2009;18(6):920-926.	301 (23.2)
8	Nellans K, Kowalski E, Chung K. The Epidemiology of Distal Radius Fractures. Hand Clin. 2012;28(2):113-125.	300 (30)
9	Rozental TD, Blazar PE. Functional outcome and complications after volar plating for dorsally displaced, unstable fractures of the distal radius. J Hand Surg Am. 2006;31(3):359-365.	291 (18.2)
10	Petruzzo P, Lanzetta M, Dubernard JM, et al. The International Registry on Hand and Composite Tissue Transplantation. Transplantation. 2010;90(12):1590-1594.	284 (23.7)
11	Arora R, Lutz M, Deml C, et al. A prospective randomized trial comparing nonoperative treatment with volar locking plate fixation for displaced and unstable distal radial fractures in patients sixty-five years of age and older. J Bone Joint Surg Am. 2011;93(23):2146-2153.	280 (25.5)
12	Franchignoni F, Vercelli S, Giordano A, et al. minimal clinically important difference of the disabilities of the arm, shoulder, and hand outcome measure (dash) and its shortened version (Quickdash). Journal of Orthopaedic & Sports Physical Therapy. 2014;44(1):30-39.doi:10.2519/jospt.2014.4893	272 (34)
13	MacDermid J, Richards R, Donner A, et al. Responsiveness of the short form-36, disability of the arm, shoulder, and hand questionnaire, patient-rated wrist evaluation, and physical impairment measurements in evaluating recovery after a distal radius fracture. J Hand Surg Am. 2000;25(2):330-340.	272 (12.4)
14	Chung KC, Shauver MJ, Birkmeyer JD. Trends in the United States in the treatment of distal radial fractures in the elderly. J Bone Joint Surg Am. 2009;91(8):1868-1873.	271 (20.8)
15	Coleman S. Hand rejuvenation with structural fat grafting. Plast Reconstr Surg. 2002;110(7):1731-1744.	237 (11.9)
16	Peolsson, Rune Hedlund, Birgitta Ob A. Intra- and inter-tester reliability and reference values for hand strength. J Rehabil Med. 2001;33(1):36-41.	236 (11.2)
17	Bond C, Shin A, McBride M, et al. Percutaneous Screw Fixation or Cast Immobilization for Nondisplaced Scaphoid Fractures. J Bone Joint Surg Am. 2001;83(4):483-488.	225 (10.7)
18	Merrell G, Wolfe S, Slade J. Treatment of scaphoid nonunions: Quantitative meta-analysis of the literature. J Hand Surg Am. 2002;27(4):685-691.	215 (10.8)
19	Young B, Rayan G. Outcome following nonoperative treatment of displaced distal radius fractures in low-demand patients older than 60 years. J Hand Surg Am. 2000;25(1):19-28.	212 (9.6)
20	Tang J. Clinical Outcomes Associated with Flexor Tendon Repair. Hand Clin. 2005;21(2):199-210.	210 (12.4)
21	Günther C, Bürger A, Rickert M, et al. Grip strength in healthy caucasian adults: reference values. J Hand Surg Am. 2008;33(4):558-565.	207 (14.8)
22	Sorensen A, Howard D, Tan W, et al. Minimal clinically important differences of 3 patient-rated outcomes instruments. J Hand Surg Am. 2013;38(4):641-649.	204 (22.7)
23	Soong M, Earp B, Bishop G, et al. Volar locking plate implant prominence and flexor tendon rupture. J Bone Joint Surg Am. 2011;93(4):328-335.	201 (18.3)
24	Haara M, Heliövaara M, Kröger H, et al. osteoarthritis in the carpometacarpal joint of the thumb. J Bone Joint Surg Am. 2004;86(7):1452-1457.	199 (11.1)
25	Chung K, Watt A, Kotsis S, et al. Treatment of unstable distal radial fractures with the volar locking plating system. J Bone Joint Surg Am. 2006;88(12):2687-2694.	195 (12.2)
26	Chang H, Chou K, Lin J, et al. Immediate effect of forearm Kinesio taping on maximal grip strength and force sense in healthy collegiate athletes. Physical Therapy in Sport. 2010;11(4):122-127.	193 (16.1)
27	Bellamy N, Campbell J, Haraoui B, et al. Dimensionality and clinical importance of pain and disability in hand osteoarthritis: Development of the Australian/Canadian (AUSCAN) Osteoarthritis Hand Index. Osteoarthritis Cartilage. 2002;10(11):855-862.	193 (9.7)
28	Davis T, Brady O, Dias J. Excision of the trapezium for osteoarthritis of the trapeziometacarpal joint: A study of the benefit of ligament reconstruction or tendon interposition. J Hand Surg Am. 2004;29(6):1069-1077.	191 (10.6)
29	Rozental T, Blazar P, Franko O, et al. Functional outcomes for unstable distal radial fractures treated with open reduction and internal fixation or closed reduction and percutaneous fixation. J Bone Joint Surg Am. 2009;91(8):1837-1846.	188 (14.5)
30	Gilpin D, Coleman S, Hall S, et al. Injectable collagenase clostridium histolyticum: a new nonsurgical treatment for Dupuytren's disease. J Hand Surg Am. 2010;35(12):2027-2038.e1.	186 (15.5)
31	Lalonde D, Bell M, Benoit P, et al. A multicenter prospective study of 3,110 consecutive cases of elective epinephrine use in the fingers and hand: the Dalhousie project clinical phase. J Hand Surg Am. 2005;30(5):1061-1067.	185 (10.9)
32	Cohen M, Kozin S. Degenerative arthritis of the wrist: proximal row carpectomy versus scaphoid excision and four-corner arthrodesis. J Hand Surg Am. 2001;26(1):94-104.	184 (8.8)
33	Arora R, Gabl M, Gschwentner M, et al. A comparative study of clinical and radiologic outcomes of unstable Colles type distal radius fractures in patients older than 70 years: nonoperative treatment versus volar locking plating. J Orthop Trauma. 2009;23(4):237-242.	176 (13.5)
34	Bellamy N, Campbell J, Haraoui B, et al. Clinimetric properties of the AUSCAN Osteoarthritis Hand Index: an evaluation of reliability, validity, and responsiveness. Osteoarthritis Cartilage. 2002;10(11):863-869.	175 (8.8)
35	Ruiz-Ruiz J, Mesa J, Gutiérrez A, et al. Hand size influences optimal grip span in women but not in men. J Hand Surg Am. 2002;27(5):897-901.	172 (8.6)
36	Mackinnon S. Pathophysiology of nerve compression. Hand Clin. 2002;18(2):231-241.	171 (8.6)
37	Stuart P, Berger R, Linscheid R, et al. The dorsopalmar stability of the distal radioulnar joint. J Hand Surg Am. 2000;25(4):689-699.	171 (7.8)
38	Crisco J, Coburn J, Moore D, et al. In vivo radiocarpal kinematics and the dart throwerʼs motion. J Bone Joint Surg Am. 2005;87(12):2729-2740.	165 (9.7)
39	Adams B, Berger R. An anatomic reconstruction of the distal radioulnar ligaments for posttraumatic distal radioulnar joint instability. J Hand Surg Am. 2002;27(2):243-251.	165 (8.3)
40	Medoff R. Essential Radiographic Evaluation for Distal Radius Fractures. Hand Clin. 2005;21(3):279-288.	164 (9.6)
41	Bertleff M, Meek M, Nicolai J. A prospective clinical evaluation of biodegradable Neurolac nerve guides for sensory nerve repair in the hand. J Hand Surg Am. 2005;30(3):513-518.	163 (9.6)
42	Johnson S, Chung K, Zhong L, et al. Risk of prolonged opioid use among opioid-naïve patients following common hand surgery procedures. J Hand Surg Am. 2016;41(10):947-957.e3.	162 (27)
43	Kanitakis J, Jullien D, Petruzzo P, et al. Clinicopathologic features of graft rejection of the first human hand allograft. Transplantation. 2003;76(4):688-693.	162 (8.5)
44	Tung T, Mackinnon S. Nerve transfers: indications, techniques, and outcomes. J Hand Surg Am. 2010;35(2):332-341.	161 (13.4)
45	Kornatz K, Christou E, Enoka R. Practice reduces motor unit discharge variability in a hand muscle and improves manual dexterity in old adults. J Appl Physiol. 2005;98(6):2072-2080.	158 (9.3)
46	SooHoo N, McDonald A, Seiler J, et al. Evaluation of the construct validity of the DASH questionnaire by correlation to the SF-36. J Hand Surg Am. 2002;27(3):537-541.	157 (7.9)
47	Lindau T, Adlercreutz C, Aspenberg P. Peripheral tears of the triangular fibrocartilage complex cause distal radioulnar joint instability after distal radial fractures. J Hand Surg Am. 2000;25(3):464-468.	157 (7.1)
48	Wiesler E, Chloros G, Cartwright M, et al. The use of diagnostic ultrasound in carpal tunnel syndrome. J Hand Surg Am. 2006;31(5):726-732.	156 (9.8)
49	Wilder F, Barrett J, Farina E. Joint-specific prevalence of osteoarthritis of the hand. Osteoarthritis Cartilage. 2006;14(9):953-957.	155 (9.7)
50	van Rijssen A, ter Linden H, Werker P. Five-year results of a randomized clinical trial on treatment in Dupuytrenʼs disease. Plast Reconstr Surg. 2012;129(2):469-477.	153 (15.3)

**Figure 1:**
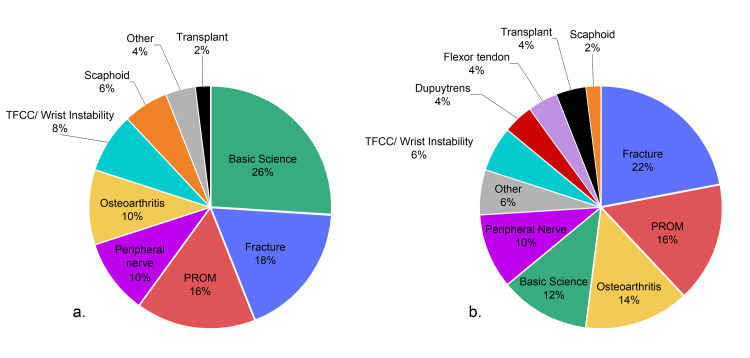
Classification of the top 50 most cited articles for all years (a) and in 2000 to 2022 (b) PROM: patient-reported outcome measures, TFCC: triangular fibrocartilage complex

Table 3 includes the article, author, and journal characteristics. The fracture was the most common article topic (n=12), as noted in Figure 1. The most common level of evidence was level IV (33%), with only four studies (11%) containing level I evidence. All publications were written in English and 78 were published by US institutions. Of the 36 institutions that produced articles within the top 50, the Mayo Clinic published the most (n=5), followed by SUNY Upstate (3) and Roosevelt Hospital (3). Table 4 contains article citations per decade. The 1980s produced the highest volume of publications (14) and had the highest mean citations per decade (428) (Table 5). The Journal of Hand Surgery (American volume) published over half (56%) of the most cited articles. The journal impact factor was not found to correlate with the number of citations (r: 0.003) or citation density (r: 0.115). The majority of authors were male with 82% of first authors and 92% of senior authors.

**Table 3:** Characteristics of the top 50 most cited articles for all years and for 2000-2022

	Top 50 Overall	Top 50 (2000-2022)
Country of Origin		
USA	39 (78%)	29 (58%)
Canada	2 (4%)	3 (6%)
Austria	2 (4%)	3 (6%)
Other	7 (14%)	15 (30%)
Authorship, Male, n		
First Author (%)	41 (82%)	38 (76%)
Senior Author (%)	44 (92%)	41 (89%)
Citation, Mean		
No. Citations (sd)	368.24 (±120.15)	233.92 (± 123.73)
Citation Density (sd)	14.98 (± 9.25)	15.34 (± 8.75)
Study Type		
Clinical	36 (72%)	44 (88%)
Basic Science	14 (28%)	6 (12%)
Level of Evidence		
I	4 (11%)	11 (25%)
II	10 (28%)	8 (18%)
III	6 (17%)	9 (20%)
IV	12 (33%)	12 (27%)
V	4 (11%)	4 (9%)
Study Design		
Cohort Study	14 (39%)	13 (30%)
Case Series	11 (31%)	11 (25%)
Randomized Controlled Trial	4 (11%)	9 (20%)
Case-Control	3 (8%)	4 (9%)
Review	2 (6%)	3 (7%)
Case Report	1 (3%)	2 (5%)
Systematic Review	-	2 (5%)
Expert Opinion	1 (3%)	-

**Table 4:** Journals and publications

Journal	No. of Publications		Impact factor
	Overall	2000-Present	
Journal of Hand Surgery (US)	28	22	2.230
Journal of Bone and Joint Surgery	9	9	5.284
Journal of Orthopaedic Trauma	2	2	2.512
Plastic and Reconstructive Surgery	2	3	4.209
Hand Clinics	2	4	1.907
Journal of Rehabilitation Medicine	1	1	2.912
Journal of Motor Behavior	1	-	1.328
Journal of Shoulder and Elbow Surgery	1	1	3.019
Journal of Orthopaedic & Sports Physical Therapy	1	1	4.751
Transplantation	1	2	4.939
Journal of Applied Physiology	1	1	3.531
Journal of Neurology Neurosurgery and Psychiatry	1	-	10.283
Osteoarthritis and Cartilage	-	3	6.576
Physical Therapy in Sport	-	1	2.365

**Table 5:** Articles and citations per decade

	1970	1980	1990	2000	2010
No. of Articles per Decade	5	14	14	13	4
Mean No. of Citations per Decade	399.6	427.9	331	357.9	284

**Figure 2:**
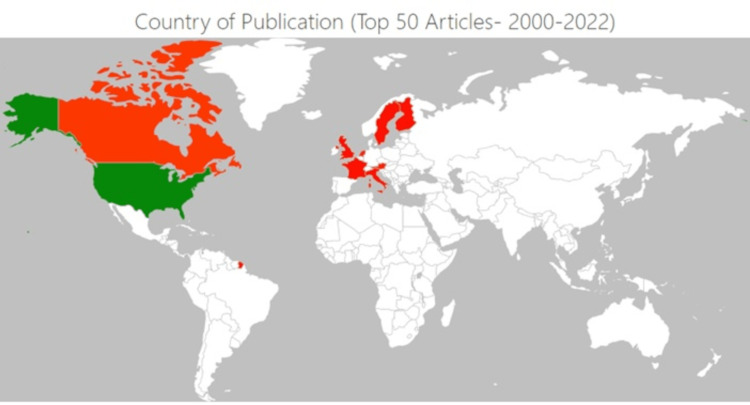
Geographic heatmap showing the countries of publication for the top 50 hand & wrist surgery articles (2000-2022)

**Figure 3:**
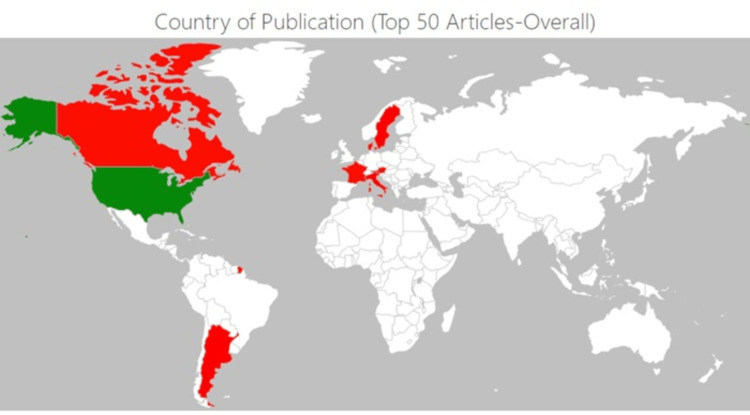
Geographic heatmap showing countries of publication for the top 50 articles (overall)

Figure 2 depicts a geographic heatmap showing the countries of publication for the top 50 hand & wrist surgery articles (2000-2022) while Figure 3 depicts a geographic heatmap showing countries of publication for the top 50 articles (overall).

The top 50 most cited hand and wrist articles from 2000-2022 were compiled in Table 2. Articles were published between 2000 and 2016. Citations ranged from 153 to 950 with a mean of 233 citations and 14.5 citation density. The most frequently discussed topic was fracture (22%), as noted in Table 4. Of clinical articles, the most common level of evidence was level IV (27%). All articles were written and English and originated from 15 countries and 12 journals. The Journal of Hand Surgery (American volume) published the most articles (44%). The impact factor of journals did not correlate with the number of citations (r: 0.129) or citation density (r: 0.145). There was a total of 42 institutions that published articles and the University Innsbruck (n=3), as well as the Washington University (n=3), published the most. Similar to the overall most cited articles, the gender of first authors (76%) and senior authors (89%) was mostly male.

Discussion

In aiming to determine the content domains of the most frequently cited articles in hand and wrist surgery, we found that articles pertaining to fracture management were cited most frequently. Fracture remained the most common article designation for papers published since 2000, indicating little change over time. Previous bibliometric analyses have been performed within various orthopedic subspecialties and have assessed content domains [11-24]. Similar to our findings, Huo et al. noted that fracture was the most common topic appearing within the 50 most cited articles in elbow surgery [23]. Kelly et al. found a prosthetic joint replacement to be the most common topic in their study analyzing the top 100 cited orthopedic surgery papers [13]. Within orthopedic trauma, distal radius fractures were reported to be the second most commonly discussed topic after hip fractures [16]. Considering the subspeciality overlap relative to fracture care in the upper extremity, it remains likely that fracture articles will continue to have a large impact with respect to citations.

With respect to the overall level of evidence contained within the most-cited article lists in hand and wrist surgery, our results indicated that level IV studies appeared most frequently. Prior studies looking at older temporal ranges of hand and wrist literature have similarly demonstrated that the most common study type is that of level IV evidence [12,18,19]. Previous studies have reported on the increase in level I evidence studies within orthopedic surgery over the recent years [12,18,19,25]. Despite the increase in impactful randomized, controlled studies on hand surgery, these papers remain far less cited than retrospective case series. This trend seems to be echoed in other fields of medicine too: A previous analysis showed that 36% of the top-cited oral surgery papers consisted of low-level evidence studies [26]. Similarly, a bibliometric analysis of 100 top-cited ophthalmology papers showed the most common level of evidence to be Level III [27]. This study has a number of limitations that should be considered. First, this study measures the impact of journals and ranks articles based on the number of citations. Citation-based metrics used exclusively do not directly measure the quality of literature. To truly assess the quality of a journal or study it is necessary to read the work, which is not always practical in studies assessing large quantities of work such as this. Low-level evidence papers receiving a high number of citations are common in other orthopedic subspecialties as well [7,28,29]. Goedderz et al. reported that 38% of the papers in their analysis of top-cited calcaneus fracture papers were of level-IV evidence [28]. Similarly, Tang et al. reported that level IV evidence studies made up 37% of the papers in the 100 top-cited anterior cruciate ligament reconstruction papers [29]. Considering the increase in level I studies over the recent years, it remains possible that papers with higher levels of evidence may permeate the frequently cited article lists.

We found that 18% of the overall top-cited and 24% of the top-cited papers since 2000 had female first authors. Previous bibliometric analyses of hand and wrist surgery publications have not reported on the gender demographics of authors, so it is difficult to compare them to historical data [7,18]. Historically, orthopedic surgery has been shown to have the lowest percentage of women and to recruit women at lower rates when compared to other surgical specialties despite an increasing number of women entering medical school [30,31]. A recent analysis of authorship in hand surgery, however, indicates an increase in the number of female authors in hand surgery research and that there has been substantial progress toward improving gender diversity in academic hand surgery over the last 14 years [31]. Despite this promising trend toward more even representation, the current bibliometric analysis found that the majority of first authors and senior authors continued to be male. As women are increasingly represented within orthopedic groups, future investigations should endeavor to assess increases in female representation among authors.

Of interest, we found no correlation between the journal impact factor and the number of citations a study receives for the most frequently published articles. Our results highlight some of the limitations surrounding the use of journal impact factors as a measure of “prestige” among authors and readers. Additional factors, such as publication immediacy or lag, ease of access to journals, self-citations in editor letters, and the use of non-source items, could also affect the impact factor of any given journal while not necessarily increasing the quality of the scientific work that is being published in it [32-37]. While a journal’s impact factor might be a useful tool to gauge its qualitative properties, authors and readers should not use it to assess the quality of individual articles and they should be aware of its inherent limitations [33,36]. Other factors outside of the inherent articles' qualities may contribute to an increased number of citations. In orthopedics and other medical fields, studies with “significant” results (ie. studies that show statistically significant results with α<0.05) are cited twice as frequently as those without significant results [38]. Self-citation, “in-group” citation, and the authority of the senior authors may also contribute to citation bias in some cases [39,40]. Additionally, the increase in social media uses to promote scholarly research may necessitate a re-evaluation of how we measure article impact [41].

This study has a number of limitations that should be considered. While a journal’s impact factor might be a useful tool to gauge its qualitative properties, authors and readers should not use it to assess the quality of individual articles and they should be aware of its inherent limitations. Impact factors measure the average possible citations per study within the journal, however, the impact factor is highly influenced by a small proportion of articles with high relative citations [33]. Therefore, a majority of these articles are not highly cited. Also, we used categories and search terms to filter the body of current literature to articles pertaining to hand and wrist surgery. This system has the potential to miss articles relating to hand and wrist surgery simply due to how articles are cataloged. Furthermore, the citations analyzed within this study were those within academic journal articles and thus do not capture any recognition articles may have received in other media such as news outlets, textbooks, podcasts, or other web-based sources. Lastly, female authorship has the potential to have been underestimated by this study, as only the first and senior authors were analyzed. Given the small sample size, this investigation was likely insufficiently powered for any formal statistical comparisons between groups.

## Conclusions

This study provides a detailed account and bibliometric analysis of the 50 most cited hand and wrist surgery publications of all time as well as the 50 most cited articles published in the twenty-first century. Frequently cited publications often contain low levels of evidence and tend to focus on topics related to fracture care. Female authors remain underrepresented. As the number of peer-reviewed journals and articles continues to increase, these data may function as a concise, modern reading list of impactful publications for hand and upper-extremity surgeons.

## Figures and Tables

**Figure 4:**
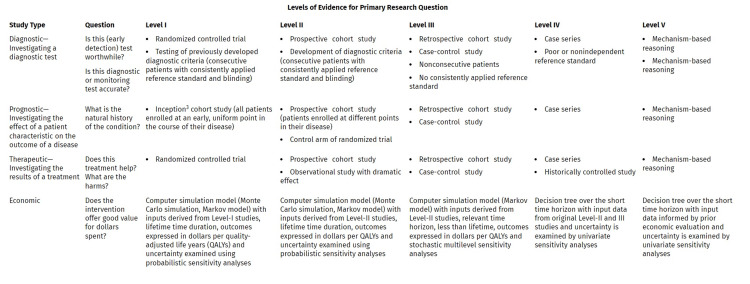
JBJS rubric that was used to analyze the level of evidence of the articles in the analysis JBJS: Journal of Bone & Joint Surgery
